# Supplementary figures and images for: Feasibility of flow-related enhancement brain perfusion MRI
Source: PLoS One. 2022 Nov 17;17(11):e0276912. doi: 10.1371/journal.pone.0276912 (PMC9671356; doi:10.1371/journal.pone.0276912)

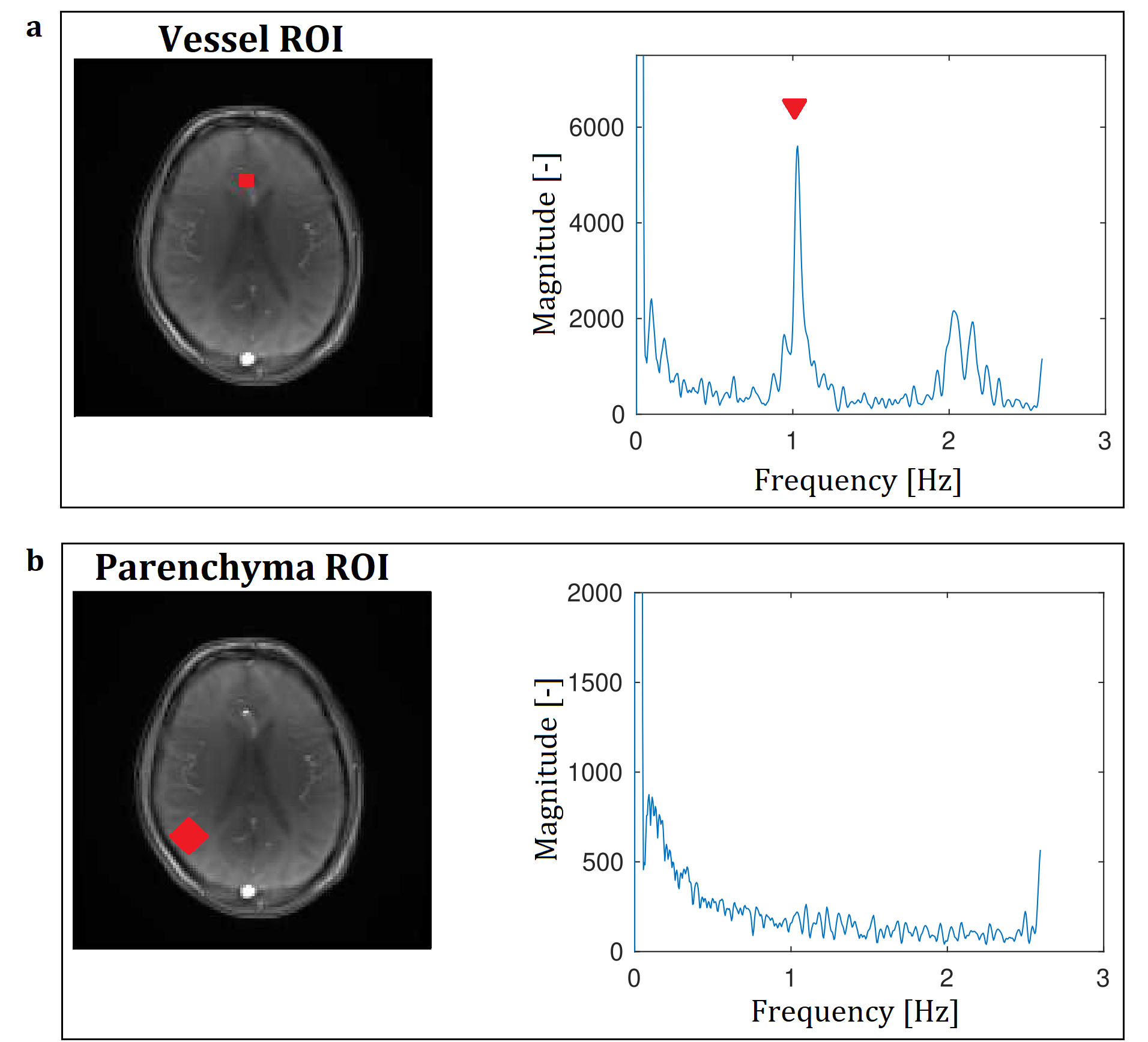

Supplement: S1 Fig — A 40° flip angle indicating a perfusion peak in the vessel (a) at the heart frequency close to 1 Hz (red triangle), but no prominent perfusion peak in the parenchyma ROI (b). (TIF) [file pone.0276912.s001.tif]

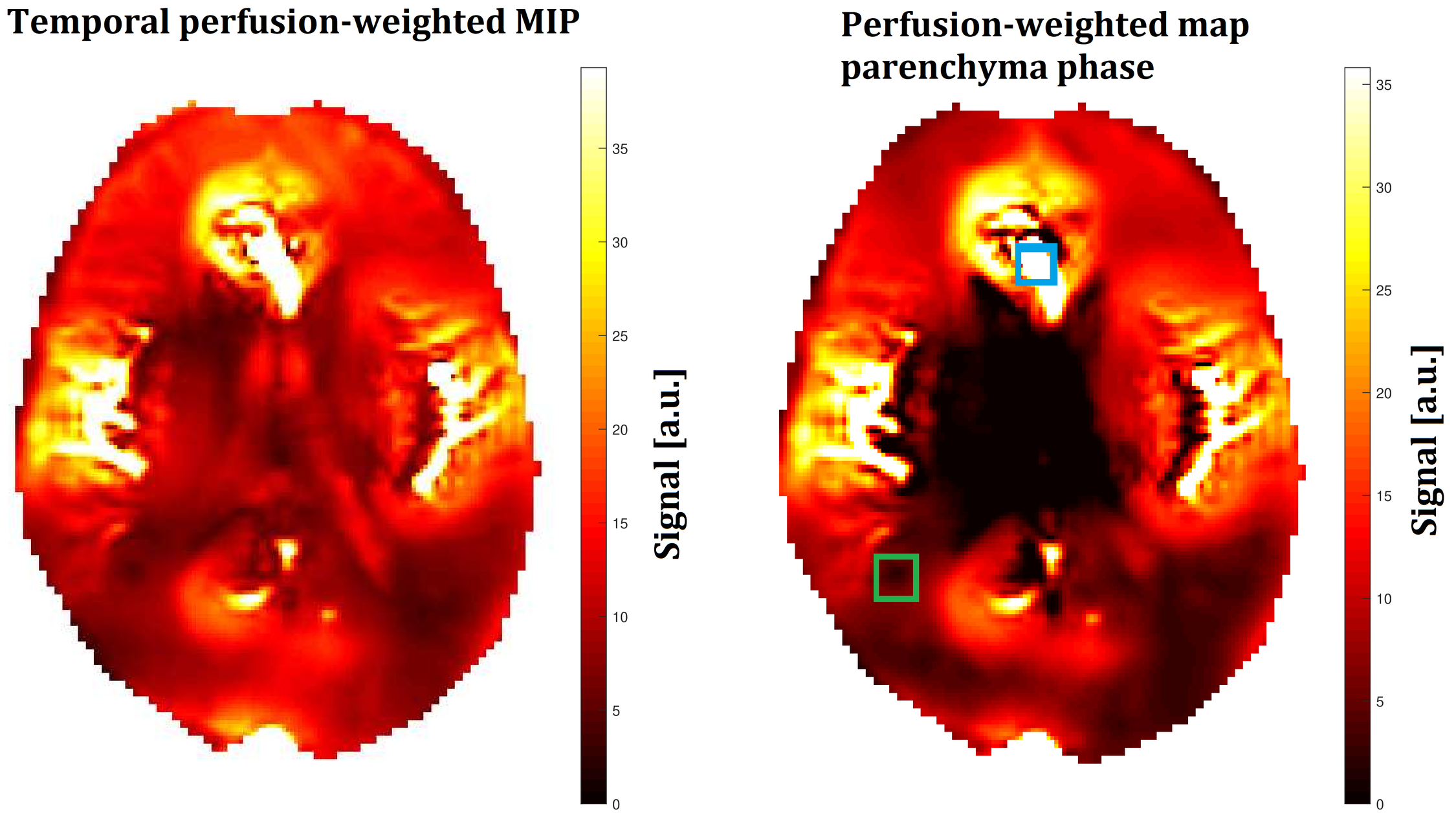

Supplement: S2 Fig — A temporal perfusion-weighted MIP is showing the maximal amplitude of the entire heart cycle for each voxel (a) and the perfusion-weighted map (b) is presenting the maximum median amplitude within the parenchyma ROI. Although showing strong perfusion contrast of 77.80 x 10−3 in the blue vessel ROI only weak corticomedullary differentiation is visible. Perfusion contrast of 5.38 x 10^−3^ in the green parenchyma ROI. (TIF) [file pone.0276912.s002.tif]

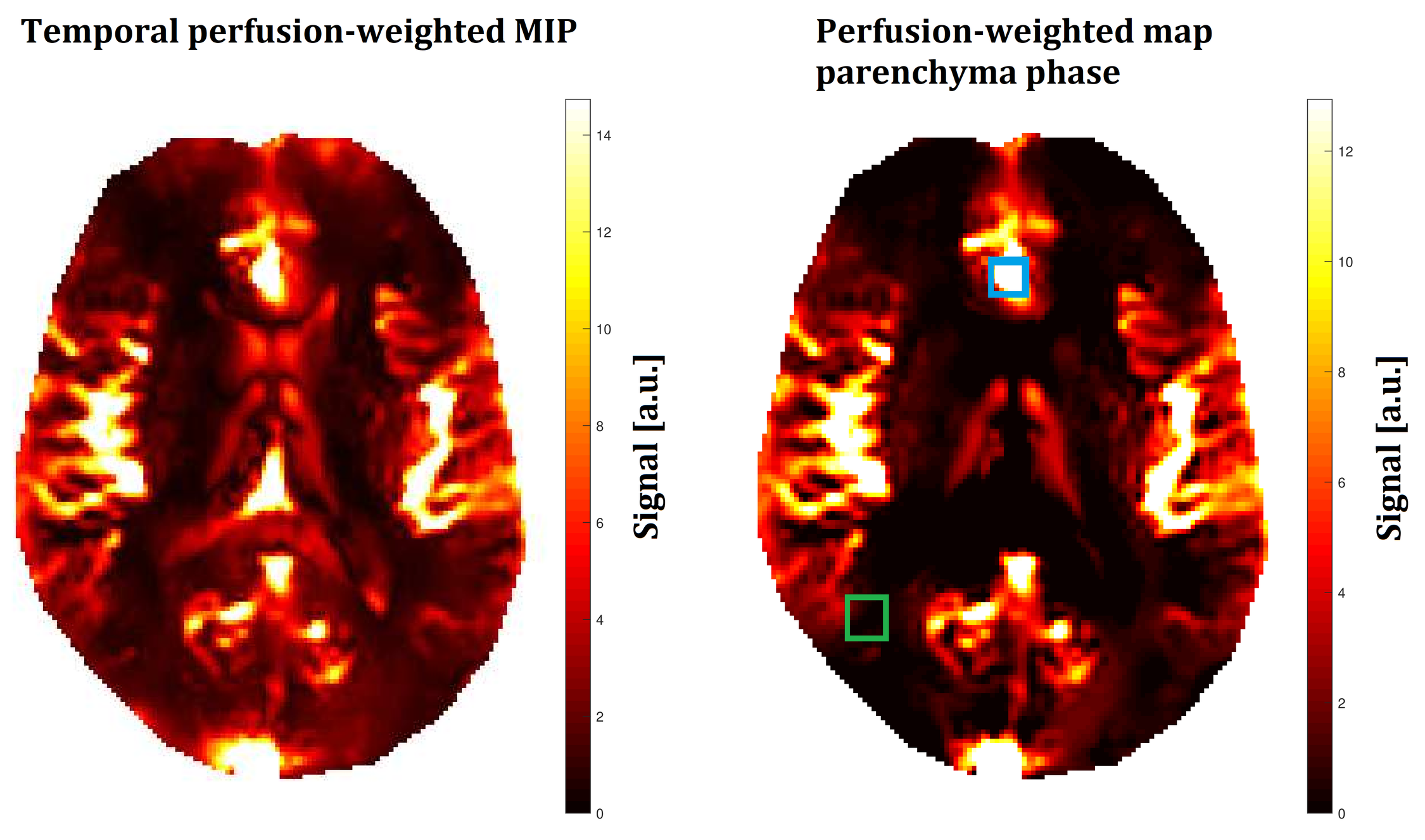

Supplement: S3 Fig — A temporal perfusion-weighted MIP is showing the maximal amplitude of the entire heart cycle for each voxel (a) and the perfusion-weighted map (b) is presenting the maximum median amplitude within the parenchyma ROI. The perfusion contrast is 27.89 x 10−3 in the blue vessel ROI and 2.96 x 10^−3^ in the green parenchyma ROI. (TIF) [file pone.0276912.s003.tif]

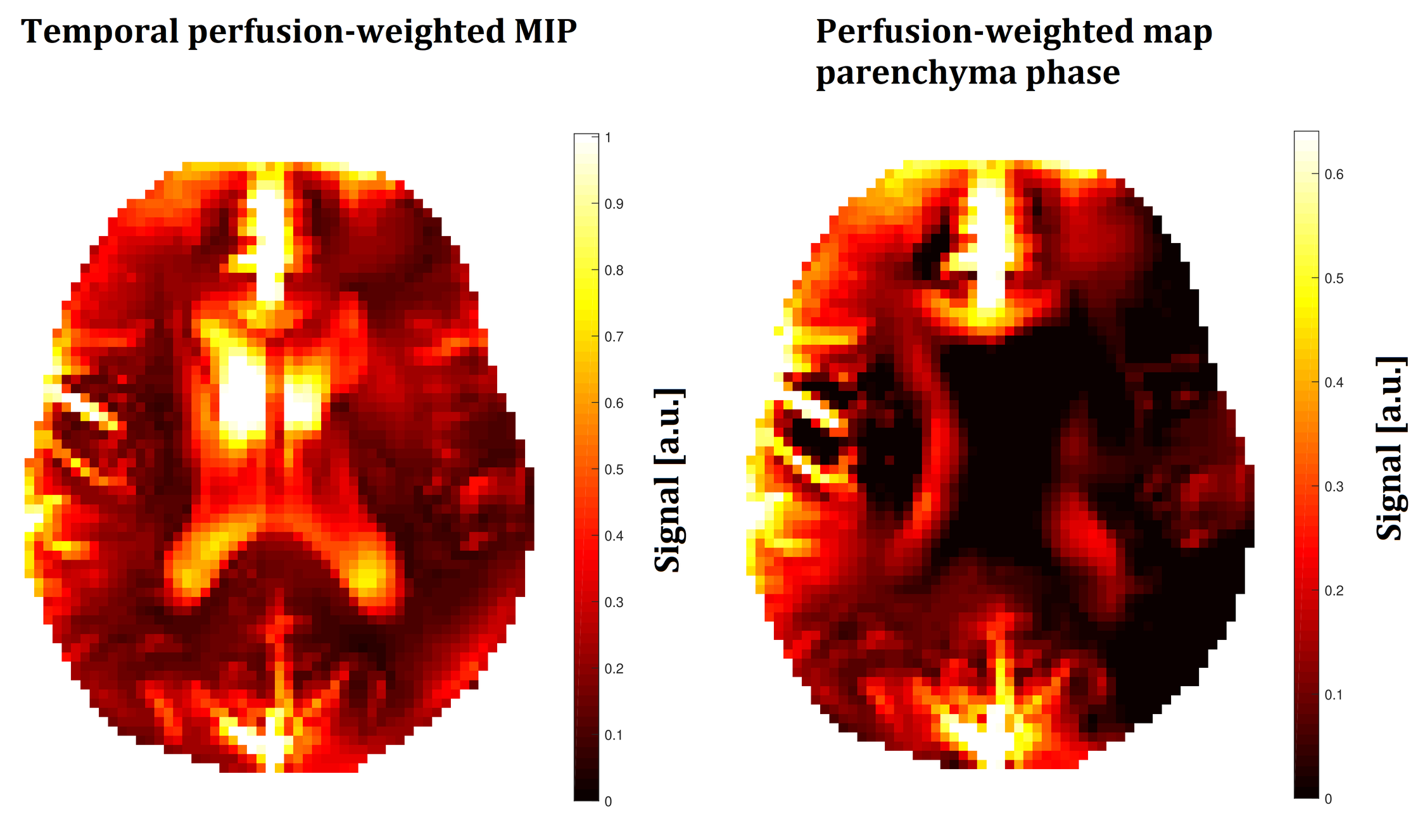

Supplement: S4 Fig — A bSSFP sequence at 1.5T with a flip angle of 55°. A temporal perfusion-weighted MIP is showing the maximal amplitude of the entire heart cycle for each voxel (left) and the perfusion-weighted map (right) is presenting the maximum median amplitude within the parenchyma ROI. Notice only minimal signal decrease in the temporal MIP, but clear demarcation of the stroke area in the parenchyma phase of the healthy hemisphere. (TIF) [file pone.0276912.s004.tif]

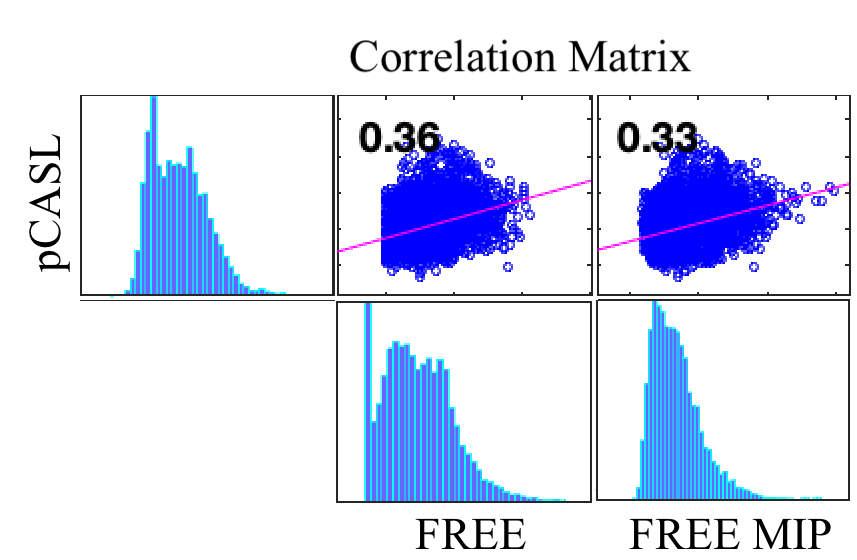

Supplement: S5 Fig — Exemplary correlation plots of a healthy participant. Whereas the FREE histogram shows many zero values, the FREE MIP presents only few. This might be caused by the differing arrival time of the pule wave between grey and white matter and no perfusion signal in the white matter yet during the phase of the perfusion-weighted FREE map. (TIF) [file pone.0276912.s005.tif]
