# Supplementary material for: Feasibility of flow-related enhancement brain perfusion MRI
Source: PLoS One. 2022 Nov 17;17(11):e0276912. doi: 10.1371/journal.pone.0276912 (PMC9671356; doi:10.1371/journal.pone.0276912)
Supplement: S1 Table — (DOCX) [file pone.0276912.s006.docx]

| Status | Stroke | Metastases |
| --- | --- | --- |
| Sequence | bSSFP | |
| Field strength [T] | 1.5 | 3 |
| Field of view [cm] | 30 x 30 | |
| Matrix size | 128 x 128 | |
| Slice thickness [mm] | 10 | |
| Echo time [ms] | 0.95 | 1.58 |
| Echo spacing [ms] | 1.95 | 3.66 |
| Flip angle [°] | 55 | 67 |
| Bandwidth [Hz/pixel] | 1000 | 501 |
| No. of channels in head coil | 32 | |
| Grappa acceleration factor | 2 | |

**S1 Table.** **MRI sequence parameters of the patients.**
