# Supplementary material for: Feasibility of flow-related enhancement brain perfusion MRI
Source: PLoS One. 2022 Nov 17;17(11):e0276912. doi: 10.1371/journal.pone.0276912 (PMC9671356; doi:10.1371/journal.pone.0276912)
Supplement: S3 Table — (DOCX) [file pone.0276912.s008.docx]

| Sequence | 2D EPI |
| --- | --- |
| Field strength [T] | 3 |
| Field of view [cm] | 34 x 34 |
| Matrix size | 128 x 128 |
| Slice thickness [mm] | 5 |
| Echo time [ms] | 25 |
| Echo spacing [ms] | 0.61 |
| Time of repetition [ms] | 4900 |
| Flip angle [°] | 90 |
| No. of channels in head coil | 32 |
| No. of measurements | 45 |
| Labelling duration [ms] | 1800 |
| Post labelling delay [ms] | 1800 |
| Bandwidth [Hz/pixel] | 1776 |

**S3 Table. pCASL perfusion parameters of the healthy.**
