# Supplementary material for: Feasibility of flow-related enhancement brain perfusion MRI
Source: PLoS One. 2022 Nov 17;17(11):e0276912. doi: 10.1371/journal.pone.0276912 (PMC9671356; doi:10.1371/journal.pone.0276912)
Supplement: S4 Table — (DOCX) [file pone.0276912.s009.docx]

| Tube voltage [kV] | 80 |
| --- | --- |
| Tube current [mA] | 150-300 |
| Gantry/Detector Tilt [°] | 0 |
| Reconstruction Diameter [mm] | 220 |
| Rotation time [s] | 0.5 |
| Acquisition interval [s] | 2 initially, then 5 |
| Contrast media flow rate [ms/s] | 5 |
| Contrast media bolus [ml] | 50 |
| NaCl-Solution bolus [ml] | 50 |

**S4 Table.** **CT perfusion parameters of the stroke patient.**
