# Supplementary material for: Feasibility of flow-related enhancement brain perfusion MRI
Source: PLoS One. 2022 Nov 17;17(11):e0276912. doi: 10.1371/journal.pone.0276912 (PMC9671356; doi:10.1371/journal.pone.0276912)
Supplement: Attachment — Submitted filename: Response to Reviewers .docx [file pone.0276912.s012.docx]

**Response to Reviewers**

PONE-D-22-14162

Feasibility of flow-related enhancement brain perfusion MRI

PLOS ONE

Dear Dr. Fujimoto

the authors would like to thank you for the precise review of the manuscript and are grateful to submit a revised version based on the reviewers' comments. We feel, that these changes improve the quality of the manuscript. A point-by-point response to each comment can be found below.

Sincerely,

Julian Glandorf

PLOS ONE

Journal Requirements:

When submitting your revision, we need you to address these additional requirements.

1. Please ensure that your manuscript meets PLOS ONE's style requirements, including those for file naming.

Answer: The style requirements were applied.

2. We note that you have indicated that data from this study are available upon request. PLOS only allows data to be available upon request if there are legal or ethical restrictions on sharing data publicly. For more information on unacceptable data access restrictions, please see http://journals.plos.org/plosone/s/data-availability#loc-unacceptable-data-access-restrictions.

In your revised cover letter, please address the following prompts:

Answer: An anonymized data set was uploaded as a Supporting Information file.

**Comments to the Author**

Reviewer #1: 

1. The presented perfusion maps are similar to the classic CBF maps. Is it possible to give quantitative values of the blood flow. Does the method allow to calculate the cerebral blood volume (CBV) based an the kinetics of the signal?

Answer: A quantification method has been developed in functional lung MRI, but has not been modified for brain perfusion yet, which is planned in a future study. However, this is quite challenging and will most likely produce a compound effect of cerebral blood volume, perfusion and CSF flow effects. The separation and proportion of these effects will have to be evaluated extensively in comparison to the ASL-method.

2. Two patients with metastasis of a melanoma were examined. Due to the content of melanin, these metastasis appear hyperintense on T1-weighted images without contrast media. Does this effect influence the perfusion contrast?

Answer: The perfusion contrast is most likely not influenced my melanin, as the perfusion contrast is generated by a subtraction so that the melanin-effect is cancelled out.

3. Fig. 4 b: Were the measurements performed before or after radiotherapy?

Answer: The measurements were performed after radiotherapy presenting lower perfusion contrast at that area: “Notice the differing perfusion contrast after radiation of the right frontal lobe in (b).”

4. In the discussion section (in line 244) the dependency of the perfusion signal on the angle of blood flow is mentioned. In perspective, is it possible to quantify the directional dependence of perfusion using the method presented (e.g. in the sense of a perfusion tensor)?

Answer: Yes, I think so. You would need to image the specimen in three imaging planes and calculate the perfusion trajectory in each voxel depending on the height of the perfusion contrast in each plane. However, this would be time consuming.

5. In the stroke patient only the basal ganglia are hypointense on CT while the M1-segment is occludet. In such a case, the vessel is usually immediately recanalized by thrombectomy without delay by MRI. This should be commented on. What was the outcome of the patient. Did you perform a thrombectomy?

Answer: In this case there were 2 emergency patients at a time. This patient had an unknown time window and was examined by MRI to gain more information, while the other patient had a thrombectomy. Afterwards this patient had a thrombectomy as well, but showed demarked infarctions as already visible in the CT and MRI prior to thrombectomy. An additional comment was placed in the figure caption.

Reviewer #2: 

Major points:

[1] “Introduction”
This study consists of three experiments. Therefore, it is better to describe the purpose/aim and motivation of each experiment in the introduction, such as the followings:
[Aim 1]
[Aim 2]
[Aim 3]

Answer: The recommendation was applied in the introduction. The three aims of the study were expressed.

[2] “Methods”
It is helpful for broad readers to add a brief explanation of what Sorensen-Dice coefficients means or how it is calculated in 1-2 sentence.

Answer: An additional sentence with explanation was added.

[3] “Results”
Please consider describing the time required for imaging in every experiment, although the authors show the required time only in a patient with an ischemic stroke.

Answer: The imaging took 230 seconds for one slice in each measurement in the healthy cohort and 127 seconds in the patient cohort. The information was added.

[4] “Discussion”
It is better to summarize the characteristics or strengths/weaknesses of the new technique compared with those of ASL and gadolinium enhancement MRI in a table.

Answer: An additional Table (Table 2) was created for comparison of the different MRI brain perfusion techniques.

[5] “Discussion”
Please add the following limitations: (1) This study confirmed the similarity of the results between the new technique and gadolinium enhancement MRI only in the condition of brain metastases and large vessel occlusion. The feasibility for other diseases such as high-grade glioma, encephalitis, vasculitis, or minor ischemic stroke is still uncertain; (2) This study did not consider the artifact effects. Some patients (i.g. with V-P shunt) would have artifacts that would affect the MR imaging.

Answer: The limitations were addressed in the discussion section.

Minor points:

[6] “Figure 2”
Please add the explanation of (c) in the figure legend.

Answer: The reference was included.

[7] “Results”
Please rewrite the subsection title “FREE vs pCASL” because this part does not show which is better or superior but the correlation/concordance of two different methods.

Answer: It was changed to “Correlation and spatial overlap of FREE and pCASL”.

[8] “Results”
It is helpful to show the scatter plot of Pearson correlation.
Answer: A plot was added as a Supplemental Figure (S6).

[9] “Results”
Please explain or define what “Dice match” means. I guess that means the “spatial overlap assessed by Sorensen-Dice coefficients,” but it should be defined in the manuscript.
Answer: An explanation is in the section “Correlation and spatial overlap between FREE and pCASL maps”

[10] “Results”
The explanation of (a)-(g) in Figure 5 should be in the figure legend, not in the main text.

Answer: The explanation was transferred into the figure legend.

All figures were uploaded to PACE and modified accordingly.
